# Acacia Senegal (Gum Arabic) Supplementation Modulate Lipid Profile and Ameliorated Dyslipidemia among Sickle Cell Anemia Patients

**DOI:** 10.1155/2019/3129461

**Published:** 2019-06-18

**Authors:** Lamis Kaddam, Imad Fadl-Elmula, Omer Ali Eisawi, Haydar Awad Abdelrazig, Amal M. Saeed

**Affiliations:** ^1^Department of Physiology, Faculty of Medicine, Neelain University, Khartoum, Sudan; ^2^Neelain Research Centre, Faculty of Medicine, Neelain University, Khartoum, Sudan; ^3^Department of Hematology, Military Hospital, Khartoum, Sudan; ^4^Department of Pediatrics, Military Hospital, Khartoum, Sudan; ^5^Department of Physiology, Faculty of Medicine, University of Khartoum, Khartoum, Sudan

## Abstract

**Background:**

Sickle cell disease (SCD) is an inherited haemolytic anemia with a variable course and severity. Knowledge of prognostic biomarkers may help in the establishment of therapeutic intervention, management, and follow-up of patients. There have been scattered reports of low high-density lipoprotein cholesterol (HDL-C) and increased triglyceride (TG) in SCD patients. In addition, TG levels have been suggested to be elevated in patients with increased endothelial activation. An increased TG level has been associated with haemolysis, vascular dysfunction, and increased prevalence of pulmonary hypertension. Gum Arabic (GA) is an edible, dried, gummy exudate from the acacia Senegal tree. Several studies on GA ingestion have shown reduced plasma cholesterol and low-density lipoprotein (LDL) concentrations in both animals and humans. We investigated GA's therapeutic potential to modulate serum lipids in patients with sickle cell anemia.

**Methods:**

This study recruited and documented secondary outcomes in 47 patients (aged 5–42 years) carrying hemoglobin SS. The patients received 30 g/day of GA for 12 weeks. Total cholesterol, TG, LDL, and HDL were measured before and after GA intake. Cobas C311 (Roche, Germany) automated chemistry analyser was used for direct determination of the values of the lipid profile.

**Results:**

GA significantly decreased total cholesterol (TC), TG, and LDL (*p* = 0.006, 0.04, and 0.02, resp.). GA showed no effect on HDL level. Baseline serum TG and LDL correlated significantly with the hydrogen peroxide (H_2_O_2_) level, which is known as an oxidative stress marker (*p* = 0.003 and 0.04, resp.). None of the lipid profile elements correlated with age.

**Conclusion:**

Our results revealed that dyslipidemia in sickle cell patients is associated with oxidative stress but not associated with age. The findings showed that GA significantly decreased TC, LDL, and TG levels, revealing a novel effect of GA, which is considered a natural dietary fibre that can modulate lipid profile in patients with sickle cell anemia.

**Trial Registration:**

This retrospective trial is registered with ClinicalTrials.gov Identifier: NCT02467257 on 3 June, 2015.

## 1. Introduction

Sickle cell disease (SCD) is a haemoglobinopathy characterised by red cell rigidity, compromised perfusion, and tissue infarction [1]. Previous studies assumed that SCD patients may develop characteristics of metabolic syndrome, presenting with hyperglycemia, hypertension, and dyslipidemia [1, 2]. Reactive oxygen species lead to defects in plasma and erythrocyte lipids [3]; therefore, oxidative stress not only is linked to chronic inflammation, but also contributes to endothelial dysfunction [4]. These two morbid processes disturb lipid homeostasis, which, in turn, may lead to atherosclerosis in SCD patients [5]. Abnormal lipid homeostasis may have the potential to alter the membrane fluidity and functions of red blood cells in individuals with SCD [6]. Lipid profile (triglyceride [TG], total cholesterol [TC], high-density lipoprotein cholesterol [HDL-C], and low-density lipoprotein cholesterol [LDL-C]) is considered a reliable assessment of coronary artery disease risk factors [7, 8]. Earlier studies in patients with SCD reported a significant increase in TG levels [9]. They also found decreased levels of TC, HDL-C, and LDC-C [3, 8, 9]. Dyslipidemia is a major concern, since it is linked with increased mortality [1, 6]. Moreover, dyslipidemia among patients with sickle cell anemia poses an uncertain threat for coronary vascular disease and pulmonary hypertension [1, 8, 10] in particular with increase longevity among sickle patients compared to the past [2].

Gum Arabic (GA) is an edible, dried, gummy exudate from the stems and branches of Acacia Senegal and Acacia Seyal. Oral intake of GA has been shown to provide several health benefits [11], such as prebiotic effects. GA is considered an antioxidant and cytoprotective agent [12–14]. Reports on the effects of GA on lipid metabolism in clinical trials and animal experiments are inconsistent [13, 15, 16]. Several studies have reported that GA ingestion reduces plasma cholesterol and LDL concentrations in humans, but the effect is mild [16–18]. Other studies showed no effect of GA on the lipid profile [19, 20].

Earlier we reported the effect of GA as a fetal hemoglobin-inducing agent and potent antioxidant agent among SCA [21, 22]. Our aim in this report is to investigate the effect of GA ingestion on the serum lipid profile among sickle cell patients.

## 2. Patients and Methods

This was a Phase II, single-arm trial carried out for investigating the effect of GA as a lipid-lowering agent among sickle cell patients. Details of entry criteria and recruitment were presented previously [21].

## 3. Gum Arabic Administration

GA in powder form is a 100% natural extract powder produced mechanically from the wildly grown acacia Senegal tree with a particle size less than 210 *μ*m. GA was provided from Dar Savanna Ltd., Khartoum, Sudan. The properties and composition of GA have been listed elsewhere [23]; furthermore, the GA dose and administration were described in detail in a previous report [21].

Blood samples were collected before administering GA and after 12 weeks, with 2 ml in an EDTA container and 2 ml in a plain container. TC, TG, LDL, and HDL were measured before and after GA intake. Cobas C311 (Roche, Germany) automated chemistry analyser was utilized for directly determining the values of the lipid profile. The hydrogen peroxide level was determined calorimetrically using a method developed by Fossati et al. [24].

Data were analysed using SPSS version 20. Paired-samples'* t*-test was selected for comparing the pre- and postintervention results. Pearson correlation was chosen to find the correlation between different parameters, and* p*-values ≤ 0.05 were considered significant.

## 4. Results

Forty-seven patients were enrolled. All were Sudanese and aged 5–42 years; 23 were males (Table 1).

**Figure 1:**
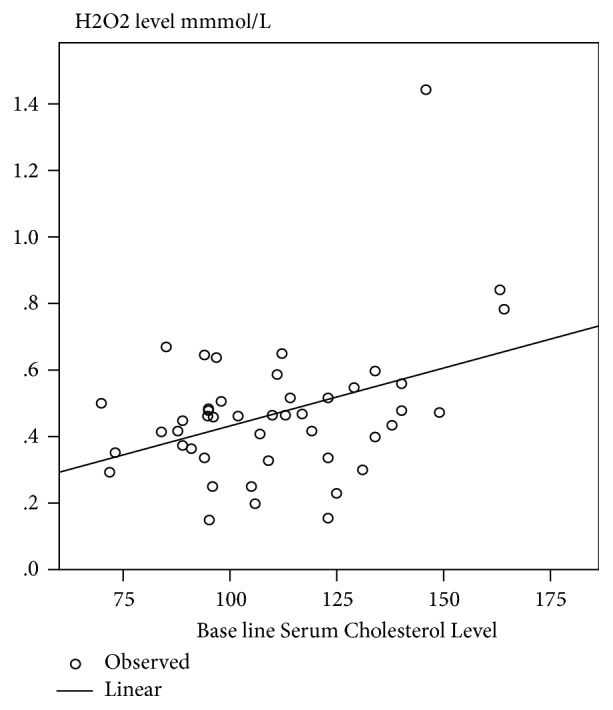
Linear regression between H2O2 and Cholesterol level (r^2^=149, P=0.008).

**Figure 2:**
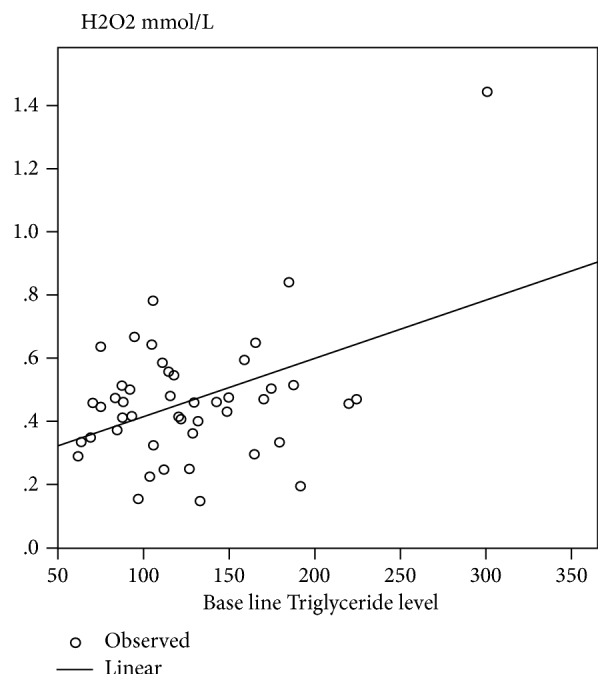
Linear regression between H_2_O_2_ and Triglycerides level (r2=185, P=0.003).

Oral GA intake significantly decreased the levels of TC, TG, and LDL (Table 2). We observed significant positive correlations between the baseline TC and H_2_O_2_ (Figure 1). We also found a significant correlation between TG and H_2_O_2_ (Figure 2).

## 5. Discussion

A point mutation in the beta globin gene results in a dysfunctional red blood cell and leads to the vasculopathy that defines SCD [25]. In SCD patients, cholesterol metabolism appears dysfunctional, as evidenced by abnormal plasma cholesterol, TG, and fatty acid content, in addition to low HDL [25, 26]. To date, there is no well-established consensus among providers on the management of the complications of SCD [27]. Many therapeutic strategies have been investigated to decrease the morbidity and mortality associated with SCD. Cholesterol-lowering agents have been found to be beneficial in reducing inflammatory markers among SCD patients [28, 29]. Moreover, previous studies revealed the therapeutic role of GA among SCA patients [21]. GA significantly augmented fetal hemoglobin level and showed potent antioxidant properties that had positive impact on patients' clinical condition and disease severity [21, 22]. Report's results revealed advantageous effect of GA on Lipid profile. GA significantly lowered the level of total cholesterol, LDL, and TSG. All of them are risk factors for atherosclerosis and metabolic syndrome [7]. GA did not increase HDL level, which would be favorable for SCD patients. Our results are consistent with previous study conducted among Sudanese population; GA reduced LDL with no effect on HDL [30]. And other contradictory outcomes revealed the augmenting effect of GA on HDL among diabetic patients [31]. Recent study conducted among Sudanese SCA patients showed low HDL among all participants and considered it as risk factor for metabolic syndrome [32]. And similar results were documented from the United States [2]. This could be explained by the pathogenesis of disease itself. Low HDL among sickles could be attributed to the nature of the disease rather than external factors. SCA patients exhibited low level of HDL-bound lecithin cholesterol acyltransferase enzyme (LCAT) and ApoAI- HDL function [26].

The study revealed significant correlation between TC and TSG and H_2_O_2_ levels (Figures 1 and 2). H_2_O_2_ is considered as toxic molecule to human tissues [33]. Sickle cell erythrocytes produce twice as much superoxide, H_2_O_2_, and hydroxyl radical as compared to normal healthy controls [34]. Relation between oxidative stress and dyslipidemia has been studied in different diseases like kidney disease [35] and metabolic syndrome [36, 37]. However, previous study showed negative relationship between oxidative stress and hypocholesterolemia [3]. In this study they measure MDA as oxidative marker instead of H_2_O_2_.

GA is soluble dietary fibers with prebiotic properties [38]. Prebiotics are considered as oligo- or polysaccharides fermented by colonic bacteria to produce short chain fatty acids [39]. Prebiotics is recently considered as novel modulator of lipid profile in vivo. Prebiotic ingestion is claimed to modulate fat content and associated metabolic disorders [31, 39–41].

Several limitations should be addressed. The study is single arm since we were aiming to investigate GA efficacy as lipid modulator among SCA patients. The short trial duration withholds us to confirm the clinical significance of our results in terms of atherosclerosis pathogenesis and related mortality. The inference of current trial is GA modulated lipids profile in favorable way among SCA patients, since it decreased LDL level. On the other hand, the most significant decline was recorded on TC level since SCA patients may have normal or low TC level compared to control; GA lowering effect may not be that beneficial to SCA patients. Thus longer and multiarm studies are recommended to affirm our findings and explore therapeutic outcome of GA on lipid profile among sickles.

## 6. Conclusion

Our results have shown that dyslipidemia in sickle cell patients is associated with oxidative stress. GA significantly decreased total cholesterol, LDL, and triglycerides levels; findings discovered a novel effect of GA, which might be consumed as natural dietary fiber to modulate lipid profile in patients with SCD and other diseases associated with dyslipidemia.

## Figures and Tables

**Table 1:** Demographics and baseline characteristics.

Characteristics	Mean	SD	Median	Maximum	Minimum
Age	16.26	8.52	15	42	5
Gender	23(49%) Male				
Baseline BMI	15.51	14.65	24.9	63	9.8
S. Cholesterol (mg/dL)	109.9	23.19	107	164	70
S.TSG(mg/dL)	126.6	48.96	116	301	62
S. LDL mg/dL	64.2	22.12	59	124	27
S.HDL mg/dL	31.36	10.79	30	56	11

**Table 2:** Comparison between the mean of pre- and postintervention values of lipid profile and H_2_O_2_.

Biomarker	Baseline mean ±SD	Postintervention mean ±SD	P.V.	95% confidence interval
S. Cholesterol (mg/dL)	109.9±24	102.1±22	.006*∗∗*	*2.353-13.136*
S.TSG(mg/dL)	126.6±49	114.1± 41.9	.048*∗*	*.1376- 25.224*
S. LDL mg/dL	64.2±22.12	58.94±22.12	.025*∗*	*.6829- 9.7852*
S.HDL mg/dL	31.36±10.8	29.47±10.63	.097	*-.35852-4.1457*
S.H_2_O_2_ (mmol/L)	0.47±0.21	0.36±0.16	0.004*∗∗*	*.03798-.18202*

*∗* Difference is significant at the 0.05 level (2-tailed).

*∗∗* Difference is significant at the 0.01 level (2-tailed).

## Data Availability

The datasets used and/or analyzed during the current study are available from the corresponding author on reasonable request.

## Abbreviations

GA: Gum Arabic
SCD: Sickle cell disease
SCA: Sickle cell anemia
H2O2: Hydrogen peroxide
HbS: Sickle hemoglobin
TC: Total cholesterol
LDL: Low-density lipoprotein
HDL: High-density lipoprotein
TSG: Triglycerides.
